# Exercise as an Adjuvant Therapy for Hematopoietic Stem Cell Mobilization

**DOI:** 10.1155/2016/7131359

**Published:** 2016-03-31

**Authors:** Russell Emmons, Grace M. Niemiro, Michael De Lisio

**Affiliations:** Department of Kinesiology and Community Health, University of Illinois at Urbana-Champaign, Urbana, IL 61801, USA

## Abstract

Hematopoietic stem cell transplant (HSCT) using mobilized peripheral blood hematopoietic stem cells (HSPCs) is the only curative strategy for many patients suffering from hematological malignancies. HSPC collection protocols rely on pharmacological agents to mobilize HSPCs to peripheral blood. Limitations including variable donor responses and long dosing protocols merit further investigations into adjuvant therapies to enhance the efficiency of HSPCs collection. Exercise, a safe and feasible intervention in patients undergoing HSCT, has been previously shown to robustly stimulate HSPC mobilization from the bone marrow. Exercise-induced HSPC mobilization is transient limiting its current clinical potential. Thus, a deeper investigation of the mechanisms responsible for exercise-induced HSPC mobilization and the factors responsible for removal of HSPCs from circulation following exercise is warranted. The present review will describe current research on exercise and HSPC mobilization, outline the potential mechanisms responsible for exercise-induced HSPC mobilization, and highlight potential sites for HSPC homing following exercise. We also outline current barriers to the implementation of exercise as an adjuvant therapy for HSPC mobilization and suggest potential strategies to overcome these barriers.

## 1. Introduction

Hematopoietic stem cell transplant (HSCT) is the only curative option for many patients with hematological malignancies. Approximately 15,000 HSCTs are performed per year in the USA, and this number is expected to rise with procedural advancements that permit HSCT in previously contraindicated patients [1]. Donor hematopoietic stem/progenitor cells (HSPCs) can be collected from a variety of sources, including umbilical cord blood (CB), bone marrow (BM), and mobilized peripheral blood (mPB), for use in transplant. Presently, nearly all autologous transplants and 75% of allogeneic transplants use mPB HSPCs as their source [2, 3]. HSPC collection from mPB is advantageous because of faster recipient reconstitution compared to CB [4] and increased ease of access compared to BM. As HSPCs are usually found only in small quantities in peripheral blood, they need to be enticed into circulation, a process known as mobilization [3]. Currently, three FDA approved drugs are available for HSPC mobilization: granulocyte colony stimulating factor (G-CSF, Filgrastim), granulocyte/macrophage colony stimulating factor (GM-CSF, Sargramostim), and AMD3100 (Plerixafor) [2, 5]. Due to higher risk of adverse events with GM-CSF, G-CSF is most commonly used, with AMD3100 being used in individuals who either are or are predicted to be poor mobilizers [2]. Although G-CSF mPB has been used for HSPC collection for HSCT since the 1980s, clinically relevant barriers still exist. Importantly, between 5 and 40% of individuals are classified as “poor mobilizers” [6–13]. These individuals do not respond well to pharmacological agents; thus, collection of sufficient HSPCs for transplant from these individuals is difficult. As such, prolonged apheresis, multiple mobilization attempts, or alternative methods of HSPC collection are necessary in these individuals resulting in increased health care costs and negative psychological effects on patients. Thus, improved strategies for HSPC mobilization in the context of HSCT, particularly in poor mobilizers, are necessary.

HSPC mobilization can occur in humans without the use of pharmacological agents. For example, HSPC quantity in peripheral blood fluctuates throughout the day and is governed by circadian rhythms [14]. Similarly, acute physiological stress can rapidly and transiently increase HSPC content in peripheral blood [15]. A growing body of evidence supports the notion that exercise, a form of physiological stress, can mobilize HSPCs into circulation [16]. These data demonstrate that acute exercise-induced HSPC mobilization is transient, while the effects of exercise training on HSPC quantity remain to be fully elucidated [17]. These data combined with recent studies demonstrating the safety of performing exercise in patients undergoing HSCT support the notion that exercise may be a potential adjuvant therapy to enhance HSPC mobilization and improve HSPC collection from mPB. The present review will explore this hypothesis by outlining the evidence that exercise can increase HSPC content in circulation, the potential mechanisms responsible for exercise-induced HSPC mobilization, and current barriers to the use of exercise as an adjuvant therapy for HSPC mobilization.

## 2. HSPC Mobilization in Response to Exercise

### 2.1. Acute Exercise

HSPCs, normally located within the bone marrow, circulate in low quantities during steady state. Physiological stress, such as acute exercise, elicits HSPC mobilization from the bone marrow into peripheral circulation [18]. In the 1980s, Heal and Brightman established the first time course for progenitor cell mobilization following exercise [19]. Using a CFU-GM assay, progenitor cell concentration in peripheral blood peaked immediately following exercise, remained elevated at 15 minutes, and returned to basal levels 1 hour following exercise in sedentary subjects [19]. With the identification of phenotypic markers for HSPCs, a 2-fold increase in circulating CD34^+^ cells has been observed within 15 minutes following exercise in paradigms including an “all out” rowing test [20] and maximal cycle ergometer test [21]. Kröpfl and colleagues, utilizing a standard incremental cycling ergometer test until exhaustion, established a time course for HSPC (identified as CD34^+^/45^dim^) mobilization into peripheral circulation at 10 minutes that returns to baseline levels as early as 30 minutes after exercise [22, 23]. Work from our lab establishes a similar time course for HSPC mobilization in mice, with an increased concentration of HSPCs detected at 15 minutes that returned to basal levels 60 minutes following an acute exercise bout [24]. The 2–4-fold increase in HSPCs following exercise in humans is consistent with the G-CSF-stimulated increase in HSPCs in some [25, 26] but not all human studies [27–29]. Although no previous studies have directly compared exercise to G-CSF, the available data suggest that exercise is a less potent mobilizer than current pharmacological approaches; however, the kinetics of exercise-induced mobilization are faster. Similarly, the exercise-induced increase in T-cells, primary initiators of the graft-versus-host response [30], is less than the increase in T-cells induced by G-CSF [31]. These data suggest that exercise-induced HSPC mobilization may not exacerbate the graft-versus-host response; however, studies examining this potential in humans have not been conducted.

Age, exercise intensity, and training status may all impact HSPC mobilization following acute exercise. Investigations looking across the lifespan including prepubertal boys and [32] sedentary men older than 65 [33] observed robust increases in HSPC mobilization following exercise. Training status may impact HSPC mobilization following exercise. Bonsignore and colleagues measured HSPC mobilization in trained athletes following either half or full marathons from samples obtained immediately upon completion of the race [34]. In both full- and half-marathon runners, no change in HSPC content was observed immediately after the race; however, a significant decrease was detected 24 hours after the race [34]. These results indicate that training status and exercise intensity play a role in the recruitment of HSPCs into peripheral circulation independent of age.

### 2.2. Exercise Training

Fewer studies have investigated the effect of exercise training on circulating HSPC quantity. Further confounding the available data is the variety among training paradigms and intensities. Paradigms utilizing higher intensity exercises, such as marathon training [34] or ischemic lower limb training conditions [35], observed an increase in circulating HSPCs after training. Bonsignore and collogues observed that half- and full-marathon athletes had higher levels of circulating HSPCs than sedentary controls [34, 36]. Training paradigms utilizing lower intensity exercise such as self-reported habitual exercise [37], treadmill walking, and cycle ergometer training [35] observed no difference in basal levels of circulating HSPCs. Niño and colleagues observed no difference in healthy young subjects engaged in 6 weeks of either progressive resistance training program, cycle ergometer training, or a combination training program [38]. Similarly, Rakobowchuk and colleagues demonstrated that healthy young subjects engaged in 6 weeks of high intensity or medium intensity interval training, 3 times per week, had no differences in circulating CD34^+^ cells upon completion of the program [39]. However, participants in the moderate intensity endurance training elicited no increase in VO_2_ after 6 weeks of endurance training while only a modest increase was seen following 6 weeks of high intensity interval training [39]. It is possible that the exercise intensity for these studies was not sufficient to induce changes in circulating HSPCs. In agreement with this notion, Wang and colleagues observed an increase in circulating HSPCs, designated as CD34^+^ cells, following 5 weeks of endurance training in hypoxic or normoxic conditions [40]. Subjects from both groups had significant increases in VO_2_ and cardiac output following training, implicating exercise intensity with increase in peripheral HSPC concentrations. Studies examining the effects of exercise training on HSPC quantity within the bone marrow are even rarer. Using mouse models, 10–15 weeks of moderate intensity exercise training, 3x per week, increased HSPC content in mouse bone marrow and circulation [41, 42]. Thus, exercise training needs to be of sufficient intensity to induce increases in circulating HSPCs, and more pronounced effects may be observed within the bone marrow than in circulation. More standardized studies will need to be conducted in the future to delineate the effect of exercise training on circulating HSPCs.

## 3. Factors Responsible for Exercise-Induced HSPC Mobilization

### 3.1. Sympathetic Nervous System

The bone marrow contains a rich network of both myelinated and nonmyelinated nerves [43] that may release catecholamine that directly impacts the HSPC populations. Previous research has demonstrated that the circadian release of NE throughout the day is related to HSPC concentrations in peripheral circulation [44]. Additionally, circadian fluctuations of NE have been associated with altered HSPC function [45, 46]. Thus, correlative* in vivo* data, supported by direct evidence* in vitro*, suggest that catecholamines may participate in HSPC mobilization. HSPCs express *α*_1_-, *α*_2_-, and *β*_2_-adrenergic receptors, which is increased by G-CSF treatment [47]. Activation of adrenergic receptors on HSPCs increased expression of membrane-associated type 1 matrix metalloproteinase (MT1-MMP) and matrix metallopeptidase-9 (MMP9) [47], which are necessary for HSPC mobilization via G-CSF [48, 49]. In addition, *β*_2_-adrenergic receptor stimulation on HSPCs from norepinephrine increases the expression of glycogen synthase kinase 3-*β* (GSK3*β*) leading to increased sensitization to chemotactic signals through cytoskeletal remodeling [18]. Furthermore, NE induced a decrease in CXCL12 expression in osteoblasts and bone marrow niche cells and enhanced HSPC mobilization with G-CSF [45]. Functional data are present to support these phenotypic changes as dopamine and norepinephrine increased motility of human HSPCs* in vitro* and egress from the bone marrow in mice [47].* In vivo*, administration of NE reuptake inhibitor, desipramine, enhanced G-CSF mobilization of HSPCs into peripheral circulation [50]. Thus, activation of sympathetic nervous system (SNS) may participate in G-CSF-induced HSPC mobilization.

Although work in murine models demonstrated a role for SNS stimulation in aiding HSPC mobilization in combination with G-CSF stimulation, translation to humans has been less clear. Patients who were chronically taking NE reuptake inhibitors or *β*-blockers did not have an increase in HSPCs in peripheral blood following G-CSF stimulation [51]. Additionally, the infusion of the *β*-agonist, isoproterenol, did not increase the circulation of HSPCs in human subjects [52] and HSPCs are not increased within peripheral circulation of patients with chronically elevated NE [53]. These studies do not rule out the possibility of SNS assisting in mobilization of HSPCs but highlight the notion that the *β*-agonist infusion alone is not sufficient to mobilize HSPCs or the contribution may be negated in states of disrupted sympathetic tone. Contrary to these studies examining chronic modulation of adrenergic signaling, exercise induces transient, physiological activation of the SNS and results in an increase in plasma norepinephrine (NE) [54] and salivary alpha-amylase (sAA) [55], a biomarker of SNS activation in the central nervous system [56]. Kröpfl and colleagues observed a 10-fold increase in mean free NE concentrations that correlated with an increase in CD34^+^/45^dim  ^ HSPCs following an exercise bout in human subjects [23]. Although these human exercise data are correlative, when considered in the context of* in vitro* data presented above, they highlight the possibility that acute alterations in SNS activity may participate in the rapid mobilization of HSPCs following exercise.

### 3.2. Cytokine Related HSPC Mobilization

SNS mediated release of HSPCs seems to work in tandem with cytokine secretion within the bone marrow. HSPCs are retained within the bone marrow through the coupling of CXCR4 on HSPCs and CXCL12 present on bone marrow stromal cells including CXCL12-abundant reticular (CAR) cells and osteoblasts [57–59]. Disruption of the CXCL12/CXCR4 axis through either increased plasma CXCL12 availability or CXCR4 antagonists, such as AMD3100, results in increased HSPC mobilization [60, 61]. G-CSF and stem cell factor (SCF), secreted by bone marrow mesenchymal stromal cells (MSCs) [62], are other factors involved in HSPC mobilization. Although the precise mechanisms by which G-CSF stimulates HSPC mobilization are complex and continually being updated, G-CSF has been shown to increase the secretion of matrix metallopeptidase-9 (MMP9) from HSPCs and enhance HSPC migration [63]. Furthermore, G-CSF increases expression of *β*_2_-adrenergic receptors on HSPCs* in vitro* and increased HSPC mobilization* in vivo* [47]. However, the direct effects of G-CSF on HSPCs have been questioned. Liu et al. demonstrated that the lack of G-CSFR on HSPCs transplanted into wild type did not prevent the egress of HSPCs into peripheral circulation [64]. In addition, injecting G-CSFR-deficient bone marrow stromal cells resulted in impaired HSPC mobilization [64] suggesting that G-CSF may be acting via indirect mechanisms. Another important cytokine involved in HSPC mobilization is SCF, which is secreted by endothelial cells, fibroblasts, and MSCs [65–67]. SCF interacts with c-Kit on HSPCs and serves as a redundant pathway to stimulate motility [68, 69]. Prolonged exposure to SCF primed HSPCs (i.e., CD34^+^ cells) to move spontaneously towards a CXCL12 gradient [69]. Interestingly, the same results were obtained when exposed to IL-3 and thrombopoietin (THPO) [69]. These data suggest that several redundant pathways in the niche exist to mediate HSPC mobilization into peripheral circulation.

Previous studies have examined exercise-induced alterations in known mobilizing agents to delineate a mechanism responsible for HSPC mobilization following exercise. Indeed, acute exercise stimulates an increase in G-CSF [70], SCF [36], and CXCL12 [71] in circulation. However, the exercise increase in all of these factors was not correlated to HSPC content following exercise [36, 68, 70]. These data are somewhat surprising given that G-CSF, SCF, and CXCL12 are powerful HSPC mobilizing agents with G-CSF being the primary mobilizing agent used clinically. A likely explanation for these apparently discrepant findings is that factors produced locally, within the HSPC niche during and after exercise, produce a stronger stimulus for HSPC mobilization. Recently, we observed an increase in G-CSF, SCF, IL-3, and THPO in the secretome of bone marrow stromal cells collected from exercised mice 15 minutes after exercise which coincided with peak HSPC concentrations in peripheral circulation [24]. These data suggest that exercise increases local G-CSF production which, along with the alterations in a variety of other cytokines induced by exercise, could account for the more rapid kinetics of HSPC mobilization with exercise compared to the slow kinetics of systemic G-CSF administration. The slow kinetics of G-CSF mobilization are likely due to the multitude of cell types expressing the G-CSF receptor, low bioavailability following injection, and rapid turnover by neutrophils and kidneys [50, 72, 73]. The increase in local G-CSF production within the HSPC niche in response to exercise may bypass many of the mechanisms related to the slow kinetics of systemic G-CSF injection. Together, these data support that exercise-induced alterations in the milieu within the bone marrow niche may play a role in stimulating robust HSPC mobilization from the bone marrow following exercise.

### 3.3. Exercise-Induced Inflammation and HSPC Mobilization

Inflammation is the activation of the innate immune system by inflammatory cytokines and can be stimulated by many events such as infection, allergies, obesity, and exercise [74–76]. Acute inflammatory states, such as infection, have been shown to mobilize HSPCs [77, 78]. In these studies, systemic infection was induced in mice using* E. coli*, a potent inducer of inflammation via LPS [79]. LPS is detected by toll-like receptor 4 (TLR-4) on immune cells, and when activated, an acute inflammatory immune response is initiated [79]. HSPCs also express TLR-4 suggesting that HSPCs may be directly activated by LPS [78]. Infection has been shown to increase HSPCs in the peripheral blood, where they later mobilized to the spleen [80]. Additionally, obesity-induced chronic inflammation is associated with increased levels of inflammatory cytokines and increased HSPCs in circulation in adults [81]. The increase in HSPCs was related directly to abdominal adiposity suggesting that inflammatory factors released from adipose tissue may promote HSPC mobilization [82, 83]. Thus, HSPCs are mobilized by acute and chronic inflammatory stimuli suggesting that acute exercise-induced inflammation may be a potential mechanism responsible for HSPC mobilization.

The acute inflammatory response to exercise involves activation of a variety of inflammatory mediators and is proportional to the intensity and duration of exercise [84]. This acute response stimulates repair processes in skeletal muscle by releasing cytokines into circulation to attract immune cells to repair muscle damage [84]. One mechanism whereby exercise may increase systemic inflammation is via the release of LPS into circulation from the gastrointestinal tract by underperfusion of the gut, which leads to mucosal damage that ultimately allows for invasion of the Gram-negative bacteria [85]. Indeed, aerobic exercise, such as short maximal tests (<20 minutes) and long term endurance exercise (>1 hour), has been shown to increase LPS in plasma immediately after exercise in humans [86–88]. Since HSPCs express TLR-4, the LPS receptor, the increase in circulating LPS induced by exercise may directly activate and mobilize HSPCs, similar to an acute infection.

In addition to increasing release of LPS from the gut, exercise also induces muscle damage that results in release of inflammatory cytokines, growth factors, and chemokines from skeletal muscle [89]. A primary mediator of this inflammatory response is IL-6. The exercise-induced increase in circulating IL-6 is intensity and duration dependent and has been shown to increase up to 100-fold following exercise [89].* In vitro* treatment of HSPCs with IL-6 results in their prolonged expansion and improved transplantation capacity [90]. Thus, exercise-induced increases in IL-6 may stimulate HSPC proliferation, which would expand the HSPC pool available for mobilization. Interestingly, IL-6 causes an increase in G-CSF by stimulating T-cells to secrete G-CSF [64]. G-CSF, an anti-inflammatory cytokine that is increased following exercise [76], is a potent mobilizer of HSPC. Although HSPCs do express the G-CSF receptor, direct interaction of G-CSF with HSPCs is not necessary to induce mobilization as HSPC mobilization occurs in G-CSFR^−/−^ mice [91]. Additionally, G-CSF disrupts the adhesive interaction of very late antigen (VLA-4/VCAM1) or chemoattractive interaction of CXCL12/CXCR4, which hold HSPCs in the bone marrow niche, thus causing release into circulation [92]. Acute aerobic exercise results in an increase in systemic levels of G-CSF [23, 30, 93–95] which is released following exercise to suppress the increase in the proinflammatory cytokine IL-6 [70, 96]. In humans, aerobic (acute and downhill running) and resistance exercise increases G-CSF concentration in circulation for 24 hours with the peak occurring at 3 hours after exercise [97]. The initial exercise-induced increase in G-CSF was positively correlated with the rise in circulating HSPCs immediately after exercise [97]. Although both G-CSF and HSPCs remained elevated in peripheral blood, in this study, 24 hours after exercise, G-CSF concentration and HSPC content were not correlated after the initial increase immediately following exercise [97]. Interestingly, creatine kinase, a marker of muscle damage, was positively correlated with HSPC content in circulation 24 hours after exercise [97]. These data suggest that the initial exercise-induced increase in G-CSF may be responsible for the early stage of HSPC mobilization, while other factors related to prolonged muscle damage may be responsible for maintaining HSPCs in circulation. The early release of G-CSF after acute exercise may cause disruption in VLA-4/VCAM or CXCL12/CXC4 interactions and cause HSPC mobilization but may not be the only mechanisms responsible for increased HSPCs in peripheral circulation following exercise.

## 4. Potential Fates of Mobilized HSPCs

Mobilized HSPCs home to tissues throughout the body to participate in the repair response [98–100]. For example, HSPCs have been found in the brain and heart in response to ischemia in stroke and myocardial infarction, respectively [98, 99]. Tissue damage and the local inflammatory response following trauma, inflammation, or ischemia in peripheral tissues increase expression of chemoattractants to promote homing of the innate immune cells and HSPCs [99]. Two potent chemokines that are secreted for HSPC recruitment include monocyte chemoattractant protein-1 (MCP-1) and CXCL12. MCP-1 and CXCL12 are expressed in cerebral, myocardial, and skeletal muscle tissue following damage [98, 99, 101]. Indeed, HSPCs express the receptors CCR2 [100] and CXCR4, which specifically bind MCP-1 and CXCL12, respectively. Thus, chemoattractants produced in various tissues throughout the body may draw HSPCs out of circulation to aid in tissue repair.

HSPCs have been shown to migrate due to ischemia and inflammation. HSPCs expressing CCR2 were recruited to sites of peripheral inflammation, such as a damaged liver [100] or myocardial infarction [99], to help repair inflamed tissues [100]. During myocardial infarction, HSPCs are recruited to ischemic heart tissue to repopulate the mature immune cells to aid in repair and promote immune cell proliferation [99]. Using a mouse model of myocardial infarction (MI), Nahrendorf's group demonstrated that myeloid-biased, CCR2^+^ HSPCs increased in circulation following MI, suggesting that the influx of CCR2^+^ HSPCs recruited to cardiac tissues repopulates the myeloid cells needed for tissue repair [99]. Interestingly, the authors showed that CCR2-mediated homing of HSPCs is not specific to MI [99]. Using lipopolysaccharide (LPS) injection to induce a systemic inflammatory response also increased the amount of CCR2^+^ HSPCs in circulation, suggesting that CCR2 expressing HSPCs are broadly recruited by inflammation. Furthermore, in ischemic stroke, Scott's group showed that increased HSPCs were found in the peripheral blood that correlated to increases in CXCL12 in the serum [98, 102]. CXCL12 was also significantly increased in the brain and also correlated to the increase in HSPCs in the brain. HSPCs also respond to CXCL12 levels in the bone marrow to home back to their niche after intravenous injection in HSCT [103–105]. Thus, inflammation, whether localized to damaged tissues or systemic, attracts HSPCs to participate in resolution of inflammation and tissue repair.

HSPC homing to sites of inflammation and tissue damage may explain the transient increase in mobilized HSPCs following exercise. Exercise mobilized HSPCs may home to extramedullary sites of tissue damage and inflammation, such as skeletal muscle, following exercise to participate in repair. In order to maximize the effectiveness of exercise in an adjuvant therapy to HSPC mobilization, a better understanding of potential homing sites and mechanisms responsible for HSPC homing following exercise is necessary. A potential site of HSPC homing is skeletal muscle, which secretes a variety of cytokines/chemokines following exercise [106]. Brzoska and colleagues demonstrated that bone marrow-derived stem cells (BMDC) contributed to skeletal muscle remodeling following eccentric exercise [107]. Following injection of GFP^+^ BMDC from transgenic donor mice, wild-type C57BL/6 mice were subject to downhill running. Subsequent evaluation of skeletal muscle revealed incorporation of GFP^+^ BDMC into the regenerated skeletal muscle fibers after one week of training [107]. Similarly, damage of skeletal muscle by cardiotoxin led to increased secretion of the chemokine, CXCL12, and increased presence of CD34^+^ cells, highlighting the possibility of BMDC homing to skeletal muscle during regeneration [108]. Although BMDC are not all HSPCs, these data do suggest that, similar to innate immune cells, HSPCs may be recruited to skeletal muscle to facilitate tissue repair. In support of this notion is the finding that side population (SP) cells have been isolated from skeletal muscle [109]. SP cells are a population of cells with high dye efflux capacity that can be isolated from bone marrow, peripheral blood, and skeletal muscle and are highly enriched with HSPCs [109]. Although skeletal muscle SP cells are phenotypically distinct from bone marrow SP cells, both SP cell populations are functionally similar as they are both capable of differentiation into the hematopoietic lineages* in vitro* and regenerate the hematopoietic system upon transplantation* in vivo* [109, 110]. In addition, muscle SP cells are bone marrow derived [111]. Thus, muscle SP cells may be a population of bone marrow-derived HSPCs that have taken up residence in skeletal muscle.

Acute exercise increases the expression of HSPC chemoattractant molecules in skeletal muscle. Increases in the expression of vascular endothelial growth factor (VEGF) have been observed following an acute bout of resistance training [100, 112]. In addition, VEGF expression and protein content were increased in rodent skeletal muscle after aerobic exercise [113, 114]. Similarly, the expression of CXCL12 and angiopoietin-1, two other HSPC chemoattractants, was upregulated following exercise [40, 115]. In addition, our lab observed an increase in the gene expression for homing factors CXCL12, angiopoietin-1 (ANG1), and VEGFa 15 minutes following an acute exercise bout in mice which coincided with peak HSPC content in circulation [24]. Thus, the expression of chemoattractant molecules suggests skeletal muscle as a site for HSPC homing following exercise.

The spleen represents another potential target for HSPC homing. HSPCs are maintained in the spleen as a site of extramedullary hematopoiesis and will preferentially relocate during times when the bone marrow niche is disrupted [9, 80, 116] or during infection [117]. Additionally, HSPCs home to the spleen following bone marrow transplant [41, 118]. Recently, we observed an increase in LSK^+^ cells in the spleen 48 hours following exercise [24]. Further research is necessary to determine whether the increase in spleen HSPCs was due to increased homing of bone marrow HSPCs or exercise-induced proliferation of HSPCs residing within the spleen. Overall, these data demonstrate the spleen as a potential site for HSPC homing following acute exercise and training. Thus, any interventions to improve mobilization strategies must also consider the effects of HSPC homing to systemic tissues to increase the amount of time HSPCs remain in circulation.

## 5. Future Perspectives

**Figure 1:**
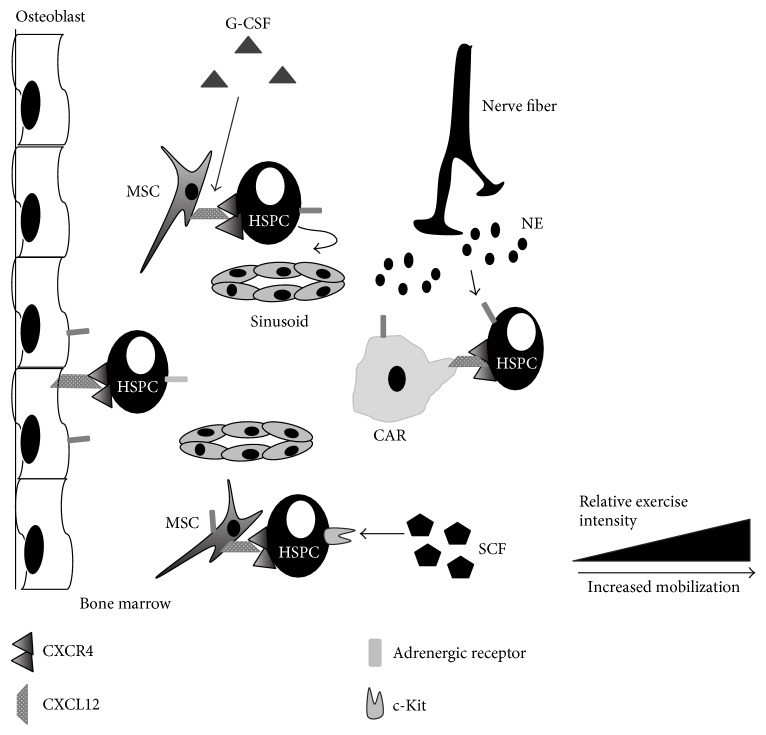
Acute exercise mobilizes hematopoietic stem and progenitors into peripheral circulation from the bone marrow niche. HSPCs receive signals from a variety of cell types including osteoblasts, mesenchymal stromal cells (MSCs), CXCL12-abundant reticular (CAR) cells, and mature hematopoietic cells throughout the bone marrow. Paracrine factors secreted by stromal cells, such as stem cell factor (SCF) or granulocyte colony stimulating factor (G-CSF), or norepinephrine by nerve fibers act to free HSPCs for entrance into peripheral circulation via sinusoidal openings. The magnitude of HSPC mobilization and paracrine factor release is increased with higher exercise intensity relative to the individual's VO_2_ max.

The present review has summarized the HSPC response to acute exercise and exercise training, the potential mechanisms responsible for the effects of exercise on HSPC mobilization, and the potential mechanisms underlying the transient nature of HSPC mobilization following exercise. Many questions remain unanswered before exercise can be recommended in clinical practice as an adjuvant therapy for HSCT. First, the precise parameters of exercise need to be better defined. The optimal mode, intensity, and duration of exercise for maximal mobilization of HSPCs need to be established, keeping in mind clinical restraints placed on HSCT patients. A recent study has suggested that traditional exercise guidelines for healthy individuals are not appropriate for patients with hematological malignancies who are candidates for HSCT [119]. Indeed, modified exercise prescription has been demonstrated to be safe and feasible in middle aged and elderly patients undergoing HSCT [120, 121]. Additionally, exercise programs in HSCT patients during the in-patient phase of treatment have been investigated, and reduced intensity programs were effective at increasing quality of life, muscle mass, and physical and emotional well-being and decreasing anxiety, fatigue, number of inpatient hospital days, and anger [122, 123]. Thus, the optimal “dose” of exercise will likely be different for healthy donors in allogeneic transplants, compared to patients mobilizing for autologous transplants. Second, a better understanding of the mechanisms responsible for exercise-induced mobilization is needed. Given the pleiotropic nature of exercise, it is likely that no single mechanism is responsible for exercise-induced HSPC mobilization and that a combination of local factors within the HSPC niche pushes HSPCs into circulation, while systemic factors in blood pull HSPCs from the marrow (Figure 1). This push/pull mechanism in response to acute exercise could be similar to what has been proposed following infection [124]. Additionally, a better understanding of these mechanisms will allow for predictions as to the interaction of exercise with currently approved pharmacological agents. For example, if exercise works via CXCL12 independent pathways, then it would be expected that exercise could synergize with AMD3100 or be beneficial in patients who are not responsive to current agents. To overcome the transient nature of HSPC mobilization, methods of inhibiting tissue inflammation and production of chemokines within skeletal muscle draw HSPCs out of circulation. These strategies could involve certain exercise modalities that minimize muscle damage or cotreatment with blocking agents that neutralize chemoattractant production in muscle. Finally, the efficacy of HSPCs mobilized by exercise needs to be established in the transplantation setting. Since exercise is a physiological stress, it is possible that exercise could cause the release of HSPCs with decreased engraftment and/or reconstitution potential and their potential effects of graft-versus-host disease. Further research is needed in transplant models to investigate the potential side effects using exercise mobilized HSPCs in HSCT.

Despite these open questions, the continued investigation of exercise as an adjuvant therapy for HSPC mobilization in HSCT is warranted due to its potential high reward with minimal risk. Indeed, a large body of literature now exists which demonstrates the feasibility and safety of exercise in patients undergoing HSCT [123, 125]. Additionally, the beneficial effects of exercise for improving physical fitness and quality of life have also been established [123, 125]. Finally, in addition to potentially enhancing HSPC mobilization, exercise preconditioning in autologous transplant patients may also optimize the stem cell niche to receive transplanted HSPCs [125]. Thus, exercise may provide a safe, feasible, low-cost approach to enhance HSPC mobilization; however, future studies directly comparing exercise against or in addition to standard pharmacological treatments and in patient populations are warranted. Whether exercise can decrease the length of treatment with mobilizing agents, decrease the required dose of mobilizing agents, or decrease the frequency of additional rounds/agents of mobilization remains unknown but warrants investigation.
